# Effectiveness and Safety of Certolizumab Pegol in Axial Spondyloarthritis in a Real-World Setting in Greece: A Sub-Analysis of the Prospective Non-Interventional CIMAX Cohort Study

**DOI:** 10.31138/mjr.33.1.162

**Published:** 2022-04-15

**Authors:** Gkikas Katsifis, Athina Theodoridou, Andreas Bounas, Panagiotis Georgiou, Petros Sfikakis, Kalliopi Fragiadaki, Theodoros Dimitroulas, Evangelia Mole, Lars Bauer, Thomas Kumke, Bengt Hoepken

**Affiliations:** 1 Rheumatology Clinic, Athens Naval Hospital, Athens, Greece,; 2 Euromedica General Clinic of Thessaloniki, Thessaloniki, Greece,; 3 Olympion Therapeutirion General Clinic, Patras, Greece,; 4 Rheumatology Unit, Agios Andreas Hospital, Patras, Greece,; 5 Laikon General Hospital, Athens, Greece,; 6 4^th^ Department of Internal Medicine, Hippokrateion General Hospital of Thessaloniki, School of Medicine, Aristotle University, Thessaloniki, Greece,; 7 Evangelismos General Hospital, Athens, Greece,; 8 UCB Pharma, Monheim am Rhein, Germany

**Keywords:** axial spondyloarthritis, TNF inhibitor, certolizumab pegol

## Abstract

**Objectives:**

We report the effectiveness and safety of certolizumab pegol (CZP) treatment in a real-world Greek axial spondyloarthritis (axSpA) population, including patients with radiographic (r-axSpA) and non-radiographic (nr-axSpA) disease.

**Methods:**

We performed a sub-analysis of the Greek cohort from CIMAX (NCT02354105), a multicentre, non-interventional cohort study that prospectively investigated CZP treatment in patients with axSpA. The primary outcome was change from baseline (CfB) in Bath Ankylosing Spondylitis Disease Activity Index (BASDAI) to Week 52.

**Results:**

Across 12 sites in Greece, 126 patients (r-axSpA: 91; nr-axSpA: 35) received ≥1 dose of CZP and were included in the Safety Set (SS), with 120 patients (r-axSpA: 86; nr-axSpA: 34) included in the Full Analysis Set (FAS). The mean (standard deviation [SD]) CfB in BASDAI at Week 52 was −3.8 (2.0) in the overall axSpA population, with numerically greater improvements observed for nr-axSpA patients compared with r-axSpA (nr-axSpA: −4.2 [2.1]; r-axSpA: −3.7 [2.0]). Improvements in the axSpA population, including r-axSpA and nr-axSpA subpopulations, were observed in key secondary and additional outcomes at Week 52. Overall, 14.3% (18/126) of patients in the axSpA population experienced ≥1 adverse event (AE). There were no serious AEs or deaths reported during the study.

**Conclusions:**

Patients with r-axSpA and nr-axSpA treated with CZP in clinical practice in Greece showed improvements in disease activity and key symptoms. CZP treatment may therefore help address the substantial health burden associated with axSpA in Greece.

## INTRODUCTION

Axial spondyloarthritis (axSpA) is a chronic, inflammatory rheumatic disease that represents a major health and financial burden for patients and society.^1,2^ The disease belongs to a group of chronic inflammatory diseases termed spondyloarthritides (SpA), which are often seen in carriers of the HLA-B27 antigen.^3–5^

The chronic inflammation associated with axSpA predominately affects the axial skeleton and entheses, with patients experiencing symptoms of chronic and severe back pain, morning stiffness and fatigue.^6^ The disease is also associated with a number of peripheral and extramusculoskeletal manifestations (EMM), including peripheral arthritis, enthesitis, acute anterior uveitis, inflammatory bowel disease (IBD) and psoriasis.^7^ These symptoms and manifestations can negatively impact patients’ quality of life and productivity.^8–10^

Patients with axSpA may also have X-ray evidence of structural damage to the sacroiliac joints; these patients are classified as having radiographic disease (r-axSpA, also known as ankylosing spondylitis), while patients who do not have definitive signs of sacroiliac joint damage on X-rays are described as having non-radiographic disease (nr-axSpA).^6^ Compared with patients with nr-axSpA, patients who have r-axSpA are more likely to be male and to have experienced a longer time since diagnosis.^6,11,12^ The prevalence of r-axSpA in the general adult population of Greece is thought to be between 0.24% and 0.29%,^13,14^ with more than 80% of these patients likely to be HLA-B27 carriers.^3^ The prevalence of nr-axSpA in Greece is unclear.

The Assessment of Spondyloarthritis international Society (ASAS) and the European Alliance of Associations for Rheumatology (EULAR) recommend that patients with axSpA receive non-steroidal anti-inflammatory drugs (NSAIDs) as first-line drug treatment, with biologic disease-modifying anti-rheumatic drugs (bDMARDs) offered as second-line options to patients with persistently high disease activity.^15^ The bDMARDs currently approved for patients with axSpA, including patients with both radiographic and non-radiographic disease, are tumour necrosis factor inhibitors (TNFi) and interleukin-17A (IL-17A) inhibitors.^6,16^ In 2014–2015, 9,279 SpA patients using pharmacy dispensed prescriptions for bDMARDs were identified in a Greek prescription database covering more than 95% of the Greek population (10,223,000 Greek citizens).^17^

The efficacy and safety of the TNFi certolizumab pegol (CZP) has been demonstrated in patients with axSpA in clinical trial settings.^12,18–20^ In addition, a recent non- interventional European study (CIMAX) was conducted to assess CZP effectiveness and safety across the axSpA disease spectrum, in a real-world setting. Primary results of the CIMAX study have been published previously.^6^ Here, we report results from a sub-analysis of the Greek cohort from CIMAX, which was conducted to assess the effectiveness and safety of CZP treatment in a real-world Greek axSpA population, including patients with r-axSpA and nr-axSpA.

## METHODS

### Study design and patients

The CIMAX study (NCT02354105) was a multicentre, non-interventional cohort study conducted between January 2015 and March 2018, that prospectively investigated the effectiveness and safety of CZP in patients with axSpA being treated in clinical practice. The full study design has previously been reported.^6^

Patients were recruited from six European countries and the study was conducted at 101 study sites. This included 12 sites in Greece located across Athens, Larisa, Maroussi, Patra and Thessaloniki.^21^ Eligible patients were required to have a clinical diagnosis of active axSpA (r-axSpA or nr-axSpA) from their treating physician and to have been newly prescribed CZP as part of their routine clinical care. During the study, patients were permitted to receive concomitant medications, including synthetic DMARDs (sDMARDs) and NSAIDs.

The decision to prescribe CZP was made by treating physicians based on the patients’ needs and in accordance with local regulations or guidelines. The dose and administration schedule for CZP treatment were selected according to the Summary of Product Characteristics (SmPC).^22^

The study was conducted in line with local legal requirements and the Declaration of Helsinki; in Greece, the study was reviewed by an Independent Ethics Committee. Prior to data collection, all patients provided written informed consent through a study-specific Patient Data Consent form. Patients were able to withdraw from the study at any time.

### Study assessments

Outcomes pertaining to effectiveness and safety were assessed up to Week 52, with clinical examinations and investigations performed by the treating physician at baseline (assessments performed up to 30 days before or 10 days after the first dose of CZP), Week 12 (Weeks 6–16), Week 24 (Weeks 17–40) and Week 52 (Weeks 41–64).

The primary outcome was the change from baseline (CfB) in Bath Ankylosing Spondylitis Disease Activity Index (BASDAI) at Week 52. The CfB in the six individual components of the BASDAI was also assessed at Week 52. The key secondary outcomes assessed were: CfB in BASDAI at Weeks 12 and 24; ASAS 20% and 40% (ASAS20/40) response at Weeks 12, 24 and 52; CfB in Bath Ankylosing Spondylitis Functional Index (BASFI) at Weeks 12, 24 and 52; CfB in Patient’s Global Assessment of Disease Activity (PtGADA) at Weeks 12, 24 and 52.

In addition, the Ankylosing Spondylitis Disease Activity Score (ASDAS, calculated with C-reactive protein [CRP] or, if unavailable, erythrocyte sedimentation rate [ESR]), total back pain and Physician’s Global Assessment of Disease Activity (PhGADA), as well as the presence of peripheral manifestations (including peripheral arthritis and enthesitis) and EMMs (including uveitis, IBD and psoriasis) were reported for Week 52. The proportion of patients achieving ASDAS clinically important improvement (ASDAS-CII; reduction of ≥1.1 from baseline), ASDAS inactive disease (ASDAS-ID; ASDAS <1.3) and ASDAS <2.1 (includes ASDAS low disease and ASDASID) were also reported at Week 52, in accordance with the ASAS/EULAR recommendations.^15^

Adverse events (AEs) were recorded when reported by a patient to their treating physician, according to the Medical Dictionary for Regulatory Activities (MedDRA^®^), version 20.1.

### Statistical analysis

Effectiveness and safety outcomes were analysed for patients in the overall Greek axSpA population, as well as by disease subgroup (Greek patients with r-axSpA or nr-axSpA) and prior TNFi exposure (Greek patients who were TNFi-naïve or TNFi-experienced). Patients who received ≥1 dose CZP were included in the Safety Set (SS); those with baseline and ≥1 post-baseline BASDAI assessment formed the Full Analysis Set (FAS) and were included in the effectiveness analyses. Patients had to have a baseline BASDAI assessment within a predefined window (up to 30 days before or 10 days after the first dose of CZP).

Effectiveness outcomes are reported using descriptive statistics. Categorical variables are reported as the percentage of responders (with 95% confidence intervals [CI]), and continuous variables are reported as mean (standard deviation [SD]).

Outcomes are reported using either observed case analysis (with no imputation for missing data), or multiple imputation (MI) or non-responder imputation (NRI). For the MI analysis, categorical age (≤/>45 years), subgroup (r-axSpA or nr-axSpA) and prior TNFi exposure (naïve or experienced) were specified as covariates and data were assumed to be missing at random. Imputed data are reported for Week 52 only. Calculation of ASDAS using MI (including ASDAS improvement and disease states), was based either on CfB in CRP or CfB in ESR; if the CRP value was missing, the observed ESR value was used and if neither were available, imputed CRP values were used. ASDAS CfB was only calculated if same-type values were available at both timepoints, eg, if both ASDAS-CRP(pre) and ASDAS-CRP(post) were available. Statistical analyses were performed using SAS^®^ (SAS Institute, Cary, NC, USA) Version 9.2.

## RESULTS

### Patient disposition and baseline characteristics

In total, 127 patients enrolled in the study from Greece. Of these, 126 started the study and 105 completed the study. The 126 patients who started the study received ≥1 dose of CZP and were therefore included in the SS. The most common reasons for study discontinuation were lack or loss of efficacy (9/126 [7.1%]), withdrawal of consent (not due to adverse events; 2/126 [1.6%]) and ‘other’ reasons (7/126 [5.5%]).

**Figure 1:**
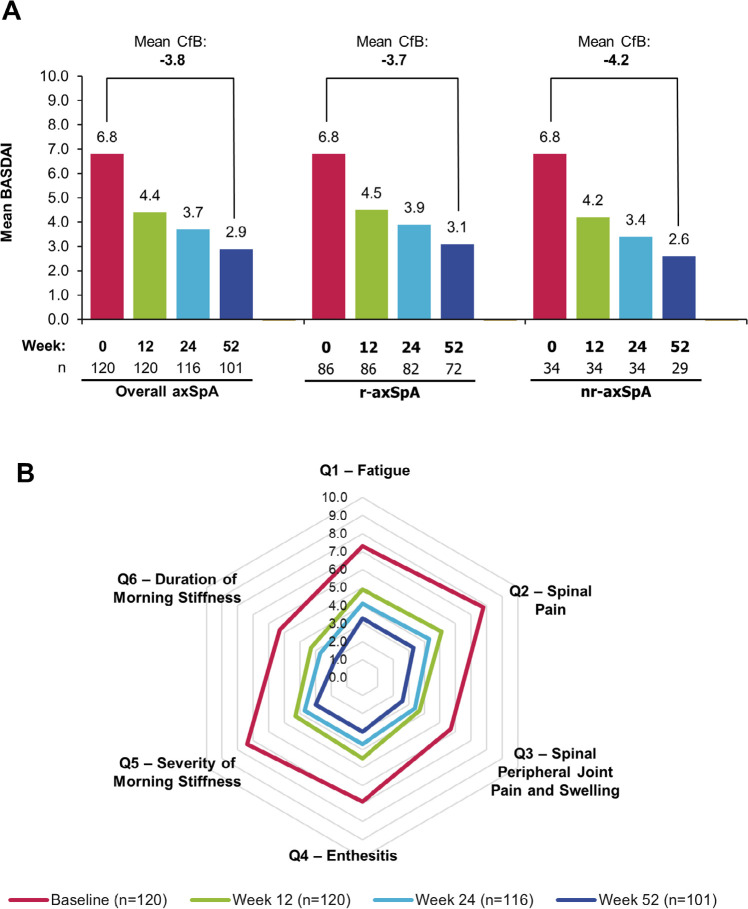
(a) BASDAI for the Greek overall axSpA, r-axSpA and nr-axSpA populations and (b) breakdown of BASDAI components for the Greek overall axSpA population. FAS (n=120). Observed case data. Mean CfB value across all patients with Week 52 BASDAI assessment is reported. axSpA: axial spondyloarthritis; BASDAI: Bath Ankylosing Spondylitis Disease Activity; CfB: change from baseline; FAS: Full Analysis Set; nr-axSpA: nonradiographic axSpA; r-axSpA: radiographic axSpA.

There were 120 patients with a baseline and ≥1 post- baseline BASDAI assessment (**Figure 1**) who were included in the effectiveness analyses (FAS). All patients in the SS and FAS had a confirmed diagnosis of r-axSpA or nr-axSpA (SS: r-axSpA: 91/126 [72.2%]; nr-axSpA: 35/126 [27.8%]; FAS: r-axSpA: 86/120 [71.7%]; nr-axSpA: 34/120 [28.3%]). Baseline demographics and disease characteristics were similar between the r-axSpA and nr-axSpA subpopulations (**Table 1**). As expected, the main differences between the r-axSpA and nr-axSpA subpopulations included the proportion of male patients (46/91 [50.5%] vs 12/35 [34.3%]), mean time since diagnosis (4.7 vs 1.3 years) and the proportion of patients with prior TNFi use (35/91 [38.5%] vs 6/35 [17.1%]). In addition, a higher proportion of patients in the nr-axSpA subpopulation had levels of CRP ≤15 mg/L (26/35 [74.3%] vs 52/91 [57.1%]) and a higher proportion had a prior history of peripheral arthritis and enthesitis (18/35 [51.4%] and 23/35 [65.7%], respectively, vs 37/91 [40.7%] and 34/91 [37.4%], respectively) compared with the r-axSpA subpopulation.

**Table 1:** Baseline demographics and disease characteristics of the Greek CIMAX population.

	**Greek cohort overall axSpA**	**Greek cohort r-axSpA**	**Greek cohort nr-axSpA**
	**(N=126)**	**(N=91)**	**(N=35)**
Age (years), mean (SD)	46.1 (12.2)	47.9 (11.7)	41.3 (12.2)
Median (range)	46.0 (22–79)	47.0 (23–79)	41.0 (22–71)
Male, n (%)	58 (46.0)	46 (50.5)	12 (34.3)
Time since diagnosis (years), mean (SD)	3.7 (5.5)	4.7 (6.1)	1.3 (2.5)
Median (range)	0.8 (0–31)	2.3 (0–31)	0.4 (0–13)
Prior TNFi use, n (%)			
Experienced	41 (32.5)	35 (38.5)	6 (17.1)
Naïve	85 (67.5)	56 (61.5)	29 (82.9)
Prior or concomitant NSAIDs, n/N (%) [a]	65/120 (54.2)	42/86 (48.8)	23/34 (67.6)
Prior or concomitant DMARDs, n/N (%) [a]	15/120 (12.5)	12/86 (14.0)	3/34 (8.8)
History of EMMs, n (%)			
Uveitis	10 (7.9)	8 (8.8)	2 (5.7)
Inflammatory bowel disease	1 (0.8)	1 (1.1)	0
Psoriasis	8 (6.3)	5 (5.5)	3 (8.6)
History of peripheral manifestations, n (%)			
Peripheral arthritis	55 (43.7)	37 (40.7)	18 (51.4)
Enthesitis	57 (45.2)	34 (37.4)	23 (65.7)
Dactylitis	5 (4.0)	1 (1.1)	4 (11.4)
Total back pain, mean	7.4 (1.9)	7.3 (1.9)	7.6 (2.0)
(SD), n [a], [b]	n=120	n=86	n=34
BASDAI, mean (SD)	6.8 (1.4)	6.8 (1.4)	6.8 (1.3)
BASFI, mean (SD)	6.0 (1.9)	6.1 (1.9)	5.7 (1.8)
ASDAS, mean (SD)	3.7 (0.8)	3.8 (0.8)	3.5 (0.7)
ASDAS <2.1, n (%)	5 (4.0)	2 (2.2)	3 (8.6)
CRP (mg/L), geometric mean	6.46	7.87	3.93
CRP level, n (%):			
≤15 mg/L	78 (61.9)	52 (57.1)	26 (74.3)
>15 mg/L	34 (27.0)	28 (30.8)	6 (17.1)
Missing	14 (11.1)	11 (12.1)	3 (8.6)
ESR (mm/h), geometric mean	19.64	19.72	19.45

SS (Greek cohort: n=126). [a] Data from the Full Analysis Set (FAS). [b] Measured using a numeric rating scale where 0 is ‘no pain’ and 10 is ‘worst possible pain’. ASDAS: Ankylosing Spondylitis Disease Activity Score; axSpA: axial spondyloarthritis; BASDAI: Bath Ankylosing Spondylitis Disease Activity Index; BASFI: Bath Ankylosing Spondylitis Functional Index; CRP: C-reactive protein; DMARD: disease-modifying anti-rheumatic drugs; EMM: extra-musculoskeletal manifestation; ESR: erythrocyte sedimentation rate; nr-axSpA: non-radiographic axSpA; NSAIDs: nonsteroidal anti-inflammatory drugs; r-axSpA: radiographic axSpA; SD: standard deviation; SS: Safety Set; TNFi: tumour necrosis factor inhibitor.

### Primary outcome

The mean (SD) CfB in BASDAI was −3.8 (2.0) for the 101/120 patients in the axSpA population who completed the Week 52 visit (**Figure 1A**). Numerically greater improvements were observed in the nr-axSpA subpopulation compared with the r-axSpA subpopulation, with nr-axSpA patients demonstrating a mean (SD) CfB of −4.2 (2.1) vs −3.7 (2.0) for patients with r-axSpA. The improvements observed when missing values were imputed (MI) were consistent with these findings, with a CfB of −3.7 (2.0) in the axSpA population, and −4.2 (2.1) and −3.5 (2.0) in the nr-axSpA and r-axSpA subpopulations, respectively (**Table 2**).

**Table 2:** Effectiveness outcomes in the Greek CIMAX population.

	**Greek cohort overall axSpA (N=120)**			**Greek cohort r-axSpA (N=86)**			**Greek cohort nr-axSpA (N=34)**		

	**Baseline**	**Week 52 (observed)**	**Week 52 (imputed)**	**Baseline**	**Week 52 (observed)**	**Week 52 (imputed)**	**Baseline**	**Week 52 (observed)**	**Week 52 (imputed)**

**BASDAI**	6.8 (1.4)	2.9 (1.9)	3.1 (2.0)	6.8 (1.4)	3.1 (1.8)	3.3 (1.9)	6.8 (1.3)	2.6 (2.0)	2.6 (2.0)
Mean (SD)	n=120	n=101		n=86	n=72		n=34	n=29	
		86.0	82.8		85.9 (61/71)	81.7		86.2 (25/29)	85.6
**ASAS20**	–		[75.7, 90.0]	–	[77.8, 94.0]	[73.1, 90.3]	–	[73.7, 98.8]	[73.1, 98.2]
% (n/N) [95% CI]		(86/100) [79.2, 92.8]							
		72.0	66.4		70.4 (50/71)	63.6		75.9 (22/29)	73.5
**ASAS40**	–		[57.6, 75.3]	–	[59.8, 81.0]	[53.0, 74.3]	–	[60.3, 91.4]	[57.9, 89.2]
% (n/N) [95% CI]		(72/100) [63.2, 80.8]							
**BASFI**	6.0 (1.9)	2.8 (1.8)	3.0 (1.9)	6.0 (1.9)	3.0 (1.8)	3.2 (1.9)	5.7 (1.8)	2.4 (1.8)	2.3 (1.7)
Mean (SD)	n=120	n=101		n=86	n=72		n=34	n=29	
**PtGADA**	7.6 (1.6)	3.1 (2.0)	3.2 (2.1)	7.5 (1.7)	3.3 (1.9)	3.4 (2.0)	7.6 (1.5)	2.7 (2.1)	2.7 (2.1)
Mean (SD)	n=120	n=100		n=86	n=71		n=34	n=29	
**ASDAS (CRP) [a]**	3.8 (0.7)	1.9 (0.9)	–	3.9 (0.8)	2.1 (0.9)	–	3.6 (0.6)	1.5 (0.7)	–
Mean (SD)	n=111	n=84		n=79	n=61		n=32	n=23	
**ASDAS-CII**	–	75 (63/84)	–		70.5 (43/61)	–		87.0 (20/23)	
% (n/N)				–			–		–
**ASDAS-ID [a]**	0 (0/113)	27.4 (23/84)	–		24.6 (15/61)	–		34.8 (8/23)	
% (n/N)				0 (0/79)			0 (0/34)		–
**ASDAS<2.1**	1.8 (2/113)	61.9 (52/84)	–		54.1 (33/61)	–		82.6 (19/23)	
% (n/N) [95% CI]				1.3 (1/79)			2.9 (1/34)		–
**Total back pain [b]**	7.4 (1.9)	3.3 (2.1)	–	7.3 (1.9)	3.3 (2.0)	–	7.6 (2.0)	3.0 (2.3)	–
Mean (SD)	n=120	n=101		n=86	n=72		n=34	n=29	
**PhGADA**	6.8 (1.4)	2.4 (1.5)	–	6.9 (1.4)	2.6 (1.6)	–	6.3 (1.6)	1.9 (1.0)	–
Mean (SD)	n=120	n=101		n=86	n=72		n=34	n=29	

FAS (n=120). Week 52 outcomes are reported using observed case analysis or multiple imputation. [a] CRP values <2 were replaced with 2 to compute ASDAS; [b] Measured using a numeric rating scale where 0 is ‘no pain’ and 10 is ‘worst possible pain’. ASAS20/40: Assessment of SpondyloArthritis international Society 20%/40% response; ASDAS: Ankylosing Spondylitis Disease Activity Score; ASDAS-CII: ASDAS clinically important improvement (change from baseline of ≥1.1); ASDAS-ID: ASDAS Inactive Disease (<1.3); axSpA: axial spondyloarthritis; BASDAI: Bath Ankylosing Spondylitis Disease Activity Index; BASFI: Bath Ankylosing Spondylitis Functional Index; CI: confidence interval; CRP: C-reactive protein; FAS: Full Analysis Set; nr-axSpA: non-radiographic axSpA; Ph/PtGADA: Physician’s/Patient’s Global Assessment of Disease Activity; r-axSpA: radiographic axSpA; SD: standard deviation.

The greatest improvement in BASDAI score between two consecutive study visits was observed between baseline and the first study visit at Week 12, with further improvements seen at subsequent study visits. The mean (SD) CfB for Weeks 12 and 24 in the axSpA population were −2.4 (1.7) and −3.1 (2.2), respectively, with similar changes observed in the r-axSpA (−2.3 [1.8] and −2.9 [2.3]) and nr-axSpA subpopulations (−2.5 [1.6] and −3.4 [2.1]) (**Figure 1A**). All six items of the BASDAI improved to a similar degree at Weeks 12, 24 and 52 for the overall axSpA population (**Figure 1B**).

### Secondary and additional variables

**Figure 2:**
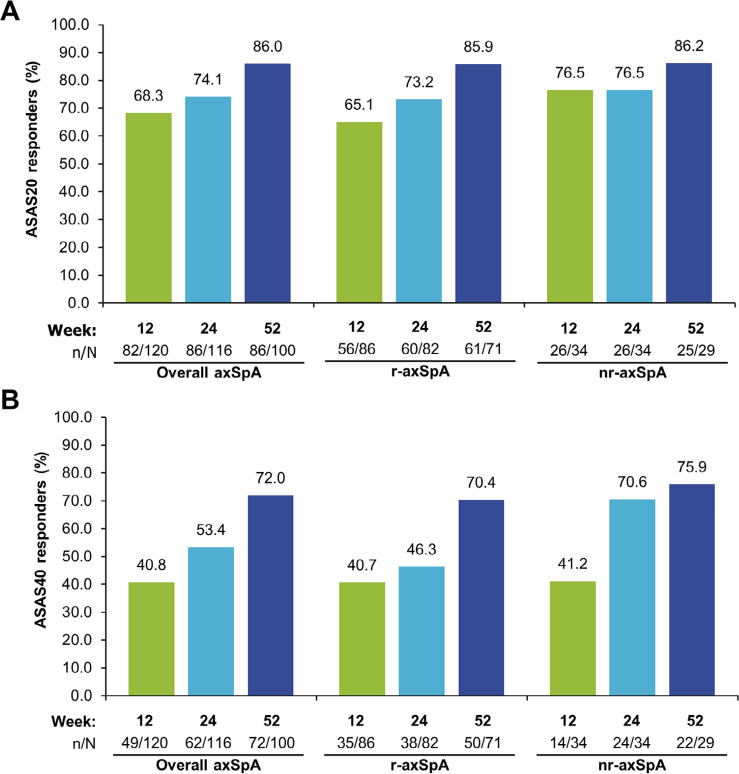
(a) ASAS20 and (b) ASAS40, in the axSpA, r-axSpA and nr-axSpA Greek CIMAX populations. FAS (n=120). Observed case data. ASAS20/40: Assessment of SpondyloArthritis international Society 20%/40% response; axSpA: axial spondyloarthritis; FAS: Full Analysis Set; nraxSpA: non-radiographic ax-SpA; r-axSpA: radiographic axSpA.

In the axSpA population overall, there were improvements in key secondary outcomes at Week 52, including in the percentage of ASAS20/40 responders (**Figure 2**). ASAS20 and ASAS40 were achieved by 86/100 (86.0%) (r-axSpA: 61/71 [85.9%]; nr-axSpA: 25/29 [86.2%]; **Figure 2A**), and 72/100 (72.0%) (r-axSpA: 50/71 [70.4%]; nr-axSpA: 22/29 [75.9%]; **Figure 2B**) of patients, respectively.

**Figure 3:**
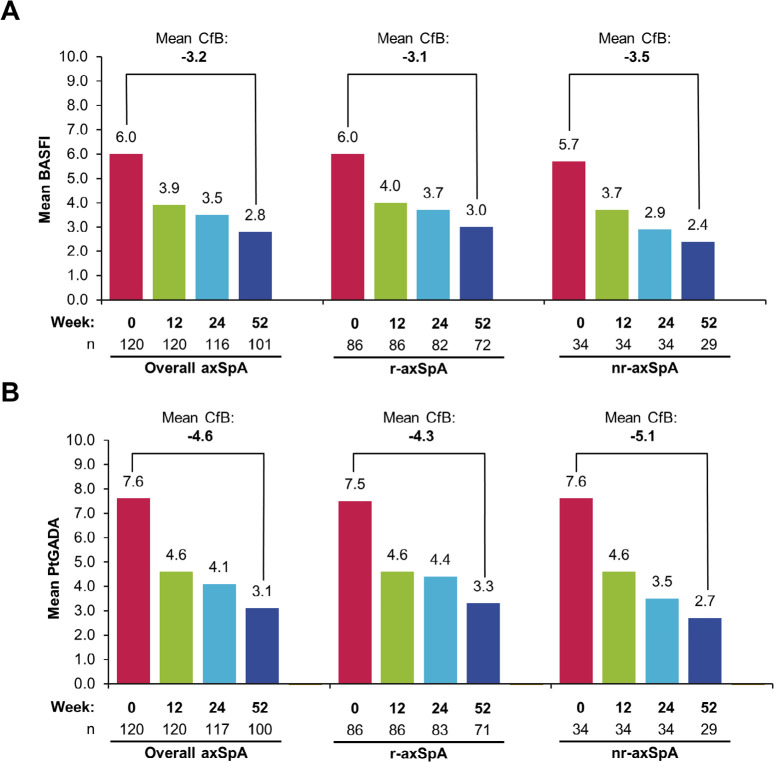
(a) BASFI and (b) PtGADA in the axSpA, r-axSpA and nr-axSpA Greek CIMAX populations. FAS (n=120). Observed case data. axSpA: axial spondyloarthritis; BASFI: Bath Ankylosing Spondylitis Functional Index; CfB: change from Baseline; FAS: Full Analysis Set; nraxSpA: non-radiographic axSpA; PtGADA: Patient’s Global Assessment of Disease Activity; r-axSpA: radiographic axSpA.

Improvements in BASFI and PtGADA were also observed over 52 weeks in the axSpA population (**Figure 3**). At Week 52, the mean (SD) CfB in BASFI was −3.2 (2.2) in the overall axSpA population, and −3.1 (2.3) and −3.5 (2.0) in the r-axSpA and nr-axSpA subpopulations, respectively (**Figure 3A**). For PtGADA, the mean (SD) CfB at Week 52 was −4.6 (2.6) for the axSpA population, and −4.3 (2.6) and −5.1 (2.6) for the r-axSpA and nr-axSpA subpopulations, respectively (**Figure 3B**). Improvements in the axSpA population, including the r-axSpA and nr-axSpA subpopulations, for additional outcomes, such as ASDAS, PhGADA and total back pain were also observed at Week 52 (**Table 2**).

Up to Week 12, 29/120 (24.2%) and 27/120 (22.5%) of axSpA patients had episodes of peripheral arthritis or enthesitis, respectively. Episodes of peripheral arthritis and enthesitis declined up to Week 52, with episodes occurring in 22/117 (18.8%) and 11/117 (9.4%) of patients between Week 12 and 24, and in 11/104 (10.6%) and 5/104 (4.8%) of patients between Week 24 and 52, respectively.

A new diagnosis of psoriasis, or one or more psoriatic flares, occurred in 2/120 (1.7%) of axSpA patients between baseline and Week 12, and in 3/117 (2.6%) of axSpA patients between Week 12 and 24. No patients were newly diagnosed with psoriasis, or experienced a psoriatic flare, between Week 24 and 52. Up to Week 12, 1/120 (0.8%) of axSpA patients were newly diagnosed with, or had one or more episodes of uveitis, with 1/104 (1.0%) newly diagnosed or experiencing one or more uveitis episodes between Week 24 and 52. No patients were newly diagnosed with uveitis, or had an episode of uveitis, between Week 12 and 24. In addition, no patients were newly diagnosed with IBD or had an IBD flare during the study.

### Outcomes by prior TNFi therapy

For the primary outcome using observed case analysis, the mean (SD) BASDAI at baseline for those in the overall axSpA population with (n=40) and without (n=80) prior TNFi exposure was 6.6 (1.4) and 6.9 (1.4), respectively. At Week 52, mean (SD) BASDAI had reduced to 2.8 (1.8) in TNFi-experienced patients (n=30) and to 3.0 (1.9) in TNFi-naïve patients (n=71) (**Table 3**), representing a mean change of −3.7 and −3.9, respectively. For the key secondary outcomes of ASAS20/40, 27/29 (93.1%) and 59/71 (83.1%) of TNFi-experienced vs TNFi-naïve patients achieved an ASAS20 response in the axSpA population at Week 52; the proportions achieving an ASAS40 response at Week 52 were 21/29 (72.4%) and 51/71 (71.8%), respectively (**Table 3**). When analysed using NRI, similar mean (SD) BASDAI responses were seen (**Table 3**). For ASAS20, 67.5% of TNFi-experienced patients and 73.8% of TNFi-naïve patients achieved a response at Week 52; for ASAS40, 52.5% and 63.8% achieved responses for each subgroup, respectively (**Table 3**).

**Table 3:** Key effectiveness outcomes in the Greek CIMAX population by TNFi treatment.

	**Greek cohort overall axSpA**			**Greek cohort overall axSpA**						
	**TNFi-naïve(N=80)**			**TNFi-experienced (N=40)**						

	**Baseline**	**Week 52 (observed)**	**Week 52 (NRI)**	**Baseline**	**Week 52 (observed)**	**Week 52 (NRI)**

**BASDAI**	6.9 (1.4)	3.0 (1.9)	3.1 (2.1) n=80	6.6 (1.4)	2.8 (1.8)	3.0 (2.0)
Mean (SD)	n=80	n=71		n=40	n=30	n=40
**ASAS20**	–	83.1 (59/71)	73.8 (59/80)	–	93.1 (27/29)	67.5 (27/40)
% (n/N) [95% CI]		[74.4, 91.8]	[64.1, 83.4]		[83.9, 100.0]	[53.0, 82.0]
**ASAS40**	–	71.8 (51/71)	63.8 (51/80)	–	72.4 (21/29)	52.5 (21/40)
% (n/N) [95% CI]		[61.4, 82.3]	[53.2, 74.3]		[56.2, 88.7]	[37.0, 68.0]

FAS (n=120). Week 52 outcomes are reported using observed case analysis. ASAS20/40: Assessment of SpondyloArthritis international Society 20%/40% response; axSpA: axial spondyloarthritis; BASDAI: Bath Ankylosing Spondylitis Disease Activity Index; CI: confidence interval; FAS: Full Analysis Set; NRI: non-responder imputation; SD: standard deviation; TNFi: tumour necrosis factor inhibitor.

### Safety

Overall, 18/126 (14.3%) of patients in the axSpA population experienced ≥1 AE, with infections and infestations, cardiac disorders and gastrointestinal disorders reported in 5/126 (4.0%), 1/126 (0.8%) and 1/126 (0.8%) of the axSpA population, respectively (**Table 4**). Of the patients experiencing AEs, 12/126 (9.5%) were considered to have experienced drug-related AEs; drug withdrawal due to AEs occurred in 9/126 (7.1%) of patients. No patients experienced a serious AE, and no patients died during the study.

**Table 4:** Safety outcomes in the Greek CIMAX population.

**MedDRA v20.1**	**Greek cohort overall axSpA (N=126)**	**Greek cohort r-axSpA (N=91)**	**Greek cohort nr-axSpA (N=35)**
**System Organ Class**			
**Preferred Term, n (%) [#]**			
Any AE	18 (14.3) [30]	15 (16.5) [25]	3 (8.6) [5]
Drug-related AEs	12 (9.5) [19]	9 (9.9) [14]	3 (8.6) [5]
Drug withdrawal due to AE	9 (7.1) [15]	7 (7.7) [12]	2 (5.7) [3]
Serious AEs	0	0	0
Infections and infestations	5 (4.0) [7]	4 (4.4) [5]	1 (2.9) [2]
Cellulitis	1 (0.8) [1]	1 (1.1) [1]	0
Tooth abscess	1 (0.8) [1]	0	1 (2.9) [1]
Tooth infection	1 (0.8) [1]	1 (1.1) [1]	0
Respiratory tract infection	1 (0.8) [1]	1 (1.1) [1]	0
Sinusitis	1 (0.8) [1]	0	1 (2.9) [1]
Upper respiratory tract infection	1 (0.8) [1]	1 (1.1) [1]	0
Urinary tract infection	1 (0.8) [1]	1 (1.1) [1]	0
Cardiac disorders	1 (0.8) [1]	1 (1.1) [1]	0
Sinus tachycardia	1 (0.8) [1]	1 (1.1) [1]	0
Gastrointestinal disorders	1 (0.8) [1]	1 (1.1) [1]	0
Abdominal distension	1 (0.8) [1]	1 (1.1) [1]	0
Deaths	0	0	0

SS (n=126). AEs were recorded according to the Medical Dictionary for Regulatory Activities v20.1. #: number of events; AE: adverse event; axSpA: axial spondyloarthritis; nr-axSpA: non-radiographic axSpA; r-axSpA: radiographic axSpA; SS: Safety Set.

## DISCUSSION

The objective of this analysis was to assess the effectiveness and safety of CZP treatment in patients with axSpA, including r-axSpA and nr-axSpA, treated during routine clinical practice in Greece.

In the Greek axSpA population, patients treated with CZP showed improvements in effectiveness outcomes over the 52-week study period. Improvements in disease activity, as measured by BASDAI, were observed in both r-axSpA and nr-axSpA patients. Both subpopulations also showed improvements in the signs and symptoms commonly associated with axSpA (pain, morning stiffness, fatigue, inflammation, and physical function), including through patient-reported outcome measures, such as PtGADA and total back pain. In addition, a low incidence of AEs and a low rate of drug withdrawal due to AEs were reported during the study.

Compared with the overall CIMAX cohort,^6^ a numerically greater improvement in the primary outcome, mean (SD) CfB in BASDAI over 52 weeks, was observed in the Greek cohort (Greece: −3.8 [2.0]; overall: −2.9 [2.3]). Responses in the Greek cohort were also higher than in the overall cohort for ASAS20/40 (Greece: ASAS20: 86.0%; ASAS40: 72.0%; overall: ASAS20: 69.7%; ASAS40: 50.5%), BASFI (Greece: −3.2 [2.2]; overall: −2.2 [2.5]) and PtGADA (Greece: −4.6 [2.6]; overall: −4.1 [2.4]). Furthermore, the incidence of AEs was lower in the Greek cohort vs the overall cohort (Greece: 14.3%; overall: 37.9%).

Possible explanations for these differences include genetic and environmental differences between Mediterranean and Northern European countries. For instance, it is possible that exposure to ultraviolet radiation as a result of milder climatic conditions in Greece may have immunosuppressive properties.^23^ Furthermore, diet plays a role in axSpA, since the disease is associated with gastrointestinal problems.^24^ The Mediterranean diet is considered to have beneficial effects on gut microbiota, which may reduce disease activity.^25^ However, as the study was not designed to compare outcomes between patients from individual countries, limited conclusions can be drawn from these comparisons and further studies are needed to understand the observed differences between the Greek and overall population.

Additionally, in this study observed case analysis TNFi-experienced patients had equal or numerically higher responses to CZP than TNFi-naïve ones. This is an unusual response pattern, as previous studies have found lower CZP response rates in TNFi-experienced patients compared to TNFi-naïve patients.^6^ This is likely explained by the way missing data were handled and higher dropout rates in the TNFi-experienced patient subgroup than the TNFi-naïve subgroup. However, analysing the data using NRI demonstrates good levels of response across both patient subgroups.

The CIMAX study was conducted at 12 clinical sites in Greece, providing the opportunity to evaluate outcomes in the Greek axSpA patient population in a real-world setting. Compared with a clinical trial, this aspect of the study design minimises issues associated with the generalisability of results to clinical practice. Another strength of CIMAX is the inclusion of patients with both r-axSpA and nr-axSpA, as there is currently limited information in the published literature about the Greek r-axSpA and nr-axSpA populations. The main limitations of this study are the lack of a comparator arm and the fact that this post-hoc analysis was not powered to formally evaluate outcomes in patients from Greece.

## CONCLUSION

In conclusion, patients with r-axSpA and nr-axSpA that were treated with CZP in clinical practice in Greece showed improvements related to disease activity and key symptoms, as well as peripheral manifestations. This study illustrates the potential of CZP treatment to address the substantial health burden associated with axSpA in Greece and supports the use of this TNFi for the Greek axSpA population in clinical practice.

## DATA SHARING STATEMENT

Data from non-interventional studies is outside of UCB’s data sharing policy. Trial registration: https://clinicaltrials.gov/ct2/show/NCT02354105?id=NCT02354105&draw=2&rank=1.
